# Instrument selection and evaluation

**DOI:** 10.1155/S1463924688000021

**Published:** 1988

**Authors:** F. L. Mitchell

**Affiliations:** The Sycamores 11 Borrowcop Lane Staffordshire Lichfield WS14 9DF UK


Journal of Automatic Chemistry, Vol. 10, No. 1 (January–March 1988), p. 3

### Instrument selection and evaluation

**F. L. Mitchell**
*The Sycamores, 11 Borrowcop Lane, Lichfield, Staffordshire WS14 9DF, UK*

Recent years have seen rapid increases in the variety, cost and complexity of instruments used in analytical chemistry. Gone are the days when choice was a simple task and any mistake did not lead to a major financial embarassment which had to be lived with for perhaps a decade. Not only is the capital investment required in the laboratory increasing, but, in the present world economic climate, the funds available are more limited and have to be wisely spent.

It is not surprising, therefore, that much attention has been given to instrument choice and evaluation, particularly by clinical laboratory workers whose large workload has led to the development of much complex and expensive automatic equipment. In 1975 colleagues in New Zealand asked the Expert Panel on Instrumentation of the International Federation of Clinical Chemistry (IFCC) if something could be done to ensure that isolated laboratories have sufficient information from manufacturers to enable an intelligent choice of equipment to be made. The Panel responded over subsequent years by producing a series of guidelines for the listing of specifications for various instrument systems. These were intended to ensure that the manufacturer provided in a standard format, all the information required by a potential customer before selecting a particular instrument for purchase. In producing their 'glossy' literature, manufacturers have a tendency to leave out important information which perhaps does not present their instrument in the best light compared with the competition. Each guideline therefore states – 'Manufacturers using the following guidelines in the description of instruments, should state that they have done so, but if such a statement is made, no information mentioned in the guidelines must be excluded unless it does not apply to the instrument'.

It is unfortunate that only very few manufacturers have made use of the guidelines and it is easy to think of many reasons for this, not all of which are laudable. To quote T. D. Geary: 'Perhaps the expense of printing new brochures could not be justified by the number of requests for the information. It becomes a circular argument; if they are not available customers will not ask for them, if they do not ask for them they will not be made available'. It would be a great service to the community if more manufacturers would use the guidelines.

A major factor in the choice of an instrument is to have access to reliable information on evaluation, and again to this our clinical colleagues have given much thought. The situation in 1984 was reviewed in *Journal of Automatic Chemistry* by Professor Haeckel (Volume 6, p. 88). It has been assumed that there will be three stages in the evaluation of an instrument.

(1) Firstly the manufacturer organizes his own evaluation of prototypes before going into production, and at this stage he generates the information necessary for the guideline specifications mentioned earlier. The US National Committee for Clinical Laboratoy Standards has produced a protocol for this work.

(2) Secondly the instrument must be evaluated, ideally, independently, in the field. This work has all too often been done in an *ad hoc* manner, at great expense to all concerned by many potential purchasers in many countries. The results are not necessarily published and certainly are not universally accepted. The European Committee for Clinical Laboratory Standards has done much work preparing guidelines for this stage of evaluation and recommends that: 'Instruments should be evaluated in more than one centre because examples of the instruments themselves may not all perform in the same manner and also because the environment in different centres will vary. Such evaluations may be of different types: in several centres independently [multiple one-centre evaluations]; or in several centres with close co-ordination [multi-centre evaluations]'. The advantages of the last approach are: '(*a*) Provision of comparable and extensive data on the reliability of new instruments from several laboratories. Less evaluation work is required in each individual laboratory since the overall pool of data is large; (*b*) A greater degree of objectivity than is likely to be achieved by multiple one-centre evaluations; (*c*) better availability of information on instrument performance; (*d*) avoidance of unnecessary replication of evaluations; (*e*) optimal use of expertise and financial resources; and (*f*) more effective stimulation of relevant instrument improvements'.
Several evaluations have now been carried out according to this concept, underlining its value and practicability. Notably for the Hitachi 737 analyser 70 000 items of data were collected in four laboratories over a four-month period and 20 routine parameters were checked on the instrument for imprecision, inaccuracy, drift effects, range limits, carry-over, practicability and dependability. All the trials have shown the advantages of the multi-centre approach in terms of time, cost and professional acceptability of results obtained.

(3) Thirdly, the so-called 'end user' needs to confirm, before placing a new instrument on routine analysis work, that the performance criteria reported from stages 1 and 2 apply to his instrument and suit his requirement. The work involved in this should not take longer than two weeks and has been covered in a document prepared by the Expert Panel of the IFCC.

